# Neonatal Glycemic Status in Infants of Diabetic Mothers in Sulaimaniyah Governorate, Iraq

**DOI:** 10.5812/ijem-164575

**Published:** 2026-01-31

**Authors:** Basta Jalil Abdalla, Adnan Muhammad Hasan, Jamal Mohammed Hussein Lateef

**Affiliations:** 1 University of Sulaimani, Sulaymaniyah, Iraq

**Keywords:** Neonatal Hypoglycemia, Diabetic Mothers, Glycemic Control, Cohort Study

## Abstract

**Background:**

Neonatal hypoglycemia is a common and serious condition in infants born to diabetic mothers (IDMs), particularly those with gestational diabetes mellitus (GDM).

**Objectives:**

This study aimed to explore neonatal glycemic status and its associated maternal and neonatal factors in babies born to diabetic mothers.

**Methods:**

A prospective cohort study was conducted in the Neonatal Intensive Care Unit of Dr. Jamal Ahmad Rashid Pediatrics Teaching Hospital and Sulaimani Maternity Teaching Hospital on 105 neonates born to mothers with type 1, type 2, or GDM. Neonatal blood glucose levels were measured at multiple time intervals (0, 1, 3, and 6 hours). Maternal and neonatal characteristics, including sociodemographic data, clinical characteristics, and delivery outcomes, were analyzed and correlated to neonatal glycemic status.

**Results:**

Most pregnant women (61%) were aged 30 - 39 years, Kurdish (92.4%), had completed high school (33.3%), lived in urban areas (69.5%), had > 3 children (58.1%), were diagnosed with GDM (87.6%), used oral medications (54.3%), had well-controlled blood sugar levels (64.8%), and experienced cesarean section (81.9%). Type 1 diabetes mellitus (T1DM) was significantly associated with neonatal blood glucose levels over time (P = 0.003). Glycemic control demonstrated a non-significant rise in blood glucose from birth to 6 hours in all patterns. Fetal weight was inversely and significantly correlated with blood glucose at birth and at 1 hour, but not at 3 or 6 hours. Gestational age showed no significant correlation with blood glucose at birth, 1, or 3 hours, but exhibited a significant correlation at 6 hours (P = 0.030).

**Conclusions:**

Maternal T1DM, more specifically, and fetal weight with gestational age were directly correlated to neonatal glycemic status in infants of diabetic mothers, but in different time periods. Thus, early monitoring and intervention for neonatal hypoglycemia are crucial, especially for neonates with poor maternal glycemic control.

## 1. Background

Diabetes mellitus (DM) in pregnancy can be pre-existing as type 1 diabetes mellitus (T1DM) or type 2 diabetes mellitus (T2DM), or could be gestational diabetes mellitus (GDM), which develops during pregnancy and typically resolves after childbirth (1). The prevalence of GDM has seen a significant rise globally, contributing to an increase in the number of infants born to diabetic mothers (IDMs). Gestational diabetes mellitus has significant long-term health implications for both the mother and the newborn (2). Neonatal glycemic status plays a crucial role in the immediate health outcomes of newborns, particularly in IDMs (3). These infants are at an elevated risk of developing neonatal hypoglycemia, hyperbilirubinemia, respiratory distress syndrome, macrosomia (excessive birth weight), and metabolic complications (4). Neonatal hypoglycemia is the most common and concerning metabolic issue that can lead to severe neurological deficits if not promptly identified and treated (5).

The mechanism behind neonatal hypoglycemia in infants of diabetic mothers is multifaceted. High maternal blood glucose levels during pregnancy lead to fetal hyperglycemia, which in turn stimulates the fetal pancreas to produce excess insulin (6). After birth, when the maternal glucose supply is abruptly removed, the neonate’s insulin remains elevated, resulting in a rapid decline in blood glucose. This imbalance between insulin secretion and glucose availability contributes to hypoglycemia in the early hours of life (24 hours post-birth), but it can persist longer in some cases (7).

The prevalence of neonatal hypoglycemia in infants of diabetic mothers varies depending on a range of maternal and neonatal factors (8). Maternal factors include the type of maternal diabetes, the severity of maternal hyperglycemia, the timing of the diagnosis, the presence of other metabolic disorders (e.g., obesity or hypertension), and the management strategies employed during pregnancy and labour (2, 9). Neonatal factors include birth weight (especially large-for-gestational-age infants), preterm birth, and low Apgar scores (9). Studies have shown that the risk of hypoglycemia in neonates born to diabetic mothers can range from 20 - 50%, significantly higher than that of neonates of non-diabetic mothers. The incidence of hypoglycemia tends to be higher in infants of mothers with poorly controlled diabetes and those with GDM diagnosed late in pregnancy (10).

Clinical signs of neonatal hypoglycemia may include jitteriness or tremors, lethargy, cyanosis, high-pitched cry, respiratory distress, seizures in severe cases, apnea, and coma in untreated severe cases (11, 12). Glycemic control is the process of managing blood glucose levels to keep them as close to the normal range as possible, without causing dangerously low blood sugar (hypoglycemia). It is a key aspect of managing diabetes to prevent long-term complications like eye, kidney, and nerve damage. Good glycemic control is typically assessed through measurements like glycated hemoglobin (HbA1c) and by monitoring blood glucose levels regularly (13). Sulaimaniyah is a major city in the Kurdistan region of Iraq. It has increasing reports of maternal diabetes with a lack of localized data on neonatal glycemic status in IDMs (14).

## 2. Objectives

Thus, this study aimed to determine the neonatal glycemic status and its associated maternal and neonatal factors in IDMs.

## 3. Methods

### 3.1. Study Design and Setting

This prospective observational cohort study was conducted on 105 delivered newborns from 105 mothers having DM during pregnancy at the Neonatal Intensive Care Unit of Dr. Jamal Ahmad Rashid Pediatric Teaching Hospital and Sulaimani Maternity Teaching Hospital, Sulaimaniyah, Iraq, from November 2024 to January 2025, using a consecutive sampling method.

### 3.2. Inclusion Criteria

Full-term and preterm neonates with a gestational age of ≥ 26 weeks who were born to mothers with pre-existing DM (T1DM or T2DM) and GDM. Both singleton and multiple births (twins and triplets) were also included.

### 3.3 Exclusion Criteria

Infants with congenital anomalies or other metabolic disorders unrelated to maternal diabetes and those with known maternal conditions that may affect glycemic status, such as thyroid dysfunction, adrenal disorders, or genetic syndromes.

### 3.4. Data Collection and Study Protocol

A standard validated questionnaire was used to collect maternal sociodemographic data (age, nationality, educational status, residency, and parity), clinical data (type of DM/diabetic management, and glycemic control), and delivery outcomes (weight before pregnancy and after delivery, gestational age, HbA1C, Apgar score, mode of delivery, gender of the infant, and birth weight). Then, neonates' blood glucose levels were measured via a heel stick using a glucometer within the first hour, 3, and 6 hours of life. Finally, the correlations between neonatal blood sugar and maternal/neonatal factors across time intervals were determined.

The glucometer used was the Accu-Chek Active system (Roche Diagnostics, Germany), which operates on the glucose oxidase-peroxidase enzymatic method. To ensure precision, internal quality control was performed daily using manufacturer-provided standard solutions at two concentration levels. The coefficient of variation (CV) for repeated glucose measurements was < 5%, consistent with laboratory performance specifications.

### 3.5. Statistical Analysis

The data were analyzed using the Statistical Package for the Social Sciences (SPSS, IBM, USA, version 26). Descriptive statistics were presented as frequencies, percentages, means, and standard deviations. The normality of continuous variables was assessed using the Shapiro-Wilk test. Because blood glucose values under hypo- and hyperglycemic conditions showed non-normal distributions, non-parametric tests were used where appropriate. Group comparisons were made using the Kruskal-Wallis test for more than two independent groups and the Mann-Whitney U test for two-group comparisons (e.g., preterm vs. full-term neonates). Spearman’s rank correlation was used to assess relationships between neonatal blood glucose and continuous maternal or neonatal variables. Repeated measurements of neonatal blood glucose (0, 1, 3, and 6 hours) were analyzed using repeated-measures ANOVA, and when normality assumptions were not met, the Friedman non-parametric test was applied. ANCOVA was used when needed to adjust for confounding factors such as gestational age and birth weight. A two-tailed P ≤ 0.05 was considered significant.

## 4. Results

### 4.1. Maternal Sociodemographic Distribution

In this cohort of 105 pregnant women, the majority (61%) were aged 30 - 39 years, Kurdish (92.4%), had completed high school (33.3%), lived in urban areas (69.5%), and had > 3 children (58.1%) (Table 1). 

**Table 1.** Maternal Sociodemographic Characteristics

Variables and Category	No. (%)
**Age (y)**	
< 20	1 (1.0)
20 - 29	28 (26.7)
30 - 39	64 (61.0)
≥ 40	12 (11.4)
**Nationality**	
Kurdish	97 (92.4)
Arabic	8 (7.6)
**Education**	
Illiterate	14 (13.3)
Primary school	33 (31.4)
Secondary school	23 (21.9)
High school	35 (33.3)
**Residency**	
Urban	73 (69.5)
Rural	32 (30.5)
**Parity**	
One child	16 (15.2)
Two children	28 (26.7)
More than three children	61 (58.1)
**Total**	105 (100)

### 4.2. Maternal Clinical Characteristics

Most pregnant women were diagnosed with GDM (87.6%), used oral medications (54.3%), and had well-controlled blood sugar levels (64.8%) (Table 2). 

**Table 2.** Maternal Clinical Data

Variables	No (%)
**Type of DM**	
Type 1	3 (2.9)
Type 2	10 (9.5)
Gestational	92 (87.6)
**Diabetes treatment**	
Diet	26 (24.8)
Oral medication	57 (54.3)
Insulin	12 (11.4)
Combination	10 (9.5)
**Glycemic control**	
Well-controlled	68 (64.8)
Moderately-controlled	24 (22.9)
Poorly controlled	13 (12.4)
**Total**	105 (100)

Abbreviation: DM, diabetes mellitus.

### 4.3. Delivery Outcomes

Most women experienced cesarean section (81.9%), and only 18.1% were delivered by standard vaginal delivery (NVD). The average pre-pregnancy weight was 75.26 ± 11.14 kg, which increased to 86.24 ± 10.73 kg before delivery. The mean gestational age at birth was 36.78 ± 1.47 weeks, and the mean HbA1C of the mothers was 6.25 ± 1.95 mmol/mol. The average Apgar score at 1 min was 8.27 ± 1.12, and at 5 min was 9.28 ± 0.70. Regarding the neonates, 61% were males, with a mean birth weight of 3.64 ± 0.68 kg. The mean length of hospital stay was 2.78 ± 4.45 days, while the mean follow-up was 13.24 ± 12.61 hours (Table 3). 

**Table 3.** Delivery Outcomes of Study Participants ^a^

Variables	Values
**Mode of delivery**	
Normal vaginal delivery	19 (18.1)
Cesarean section	86 (81.9)
**Maternal weight (kg)**	
Pre-pregnancy	75.26 ± 11.14
Before delivery	86.24 ± 10.73
HbA1C (mmol/mol)	6.25 ± 1.95
Gestational age (wk)	36.78 ± 1.47
**APGAR score (min)**	
1	8.27 ± 1.12
5	9.28 ± 0.70
**Gender**	
Male	64 (61.0)
Female	41 (39.0)
**Birth weight (kg)**	3.64 ± 0.68
**Duration of hospital stay (d)**	2.78 ± 4.45
**Follow-up duration (h)**	13.24 ± 12.61

^a^ Values are expressed as No. (%) or mean ± SD.

### 4.4. Neonatal Blood Glucose

Using non-parametric analysis, blood glucose increased over time in all groups of diabetes; however, only neonates of type 1 diabetic mothers showed a significant increase in blood glucose levels (P = 0.003). Additionally, glycemic controls among neonates were not significantly improved across all time intervals (Table 4). 

**Table 4.** Comparison of Neonatal Blood Glucose Levels Across Time Intervals According to Maternal Factors ^a^

Variables	Blood Glucose (mg/dL) Across Time Interval				P-Value
	0 h	1 h	3 h	6 h	
**Type of diabetes**					
Type 1	47.33 ± 2.89	51.57 ± 13.87	69.33 ± 26.86	84.76 ± 35.15	0.003 ^b^
Type 2	39.60 ± 12.25	55.90 ± 33.40	63.03 ± 18.04	59.16 ± 27.83	0.080
Gestational	48.45 ± 18.40	50.07 ± 13.28	60.88 ± 14.21	69.88 ± 12.51	0.158
**Glycemic control**					
Well	50.87 ± 19.26	52.29 ± 17.09	61.82 ± 15.44	69.93 ± 14.68	0.098
Moderate	45.65 ± 12.86	50.18 ± 12.92	61.82 ± 11.10	73.17 ± 13.325	0.112
Poor	33.86 ± 8.88	43.08 ± 14.27	57.85 ± 18.26	58.74 ± 20.58	0.068

^a^ Values are expressed as mean ± SD.

^b^ Significant at P < 0.05, using Kruskal-Wallis test (non-parametric).

Birth weight was inversely correlated with glucose at birth and one hour, indicating that heavier infants were more prone to early hypoglycemia. Gestational age showed a positive correlation with blood glucose at 6 hours, suggesting that more mature infants achieved higher glucose stability later in the first day of life (Table 5). 

**Table 5.** Neonatal Blood Glucose Correlations with Neonatal Factors Across Time Intervals

Variables	Time Interval (h)			
	0	1	3	6
**Birth weight (Kg)**				
r	-0.225	-0.245	-0.142	0.039
P-value	0.021 ^a^	0.012 ^a^	0.148	0.691
**Gestational age (wk)**				
r	0.108	-0.073	-0.115	0.213
P-value	0.272	0.461	0.242	0.030 ^a^

^a^ Significant at P < 0.05, using Spearman’s correlation test (non-parametric).

Regarding the comparison of neonatal blood glucose levels between preterm (< 37 weeks) and full-term (≥ 37 weeks), the mean blood glucose at birth was significantly lower in preterm infants (46.8 ± 12.4 mg/dL) compared to full-term (58.2 ± 13.7 mg/dL; P = 0.024). However, by six hours of life, this difference was no longer significant (P = 0.276). These findings suggest that preterm neonates are more prone to early hypoglycemia, but glucose levels tend to stabilize within the first few hours after birth (Table 6). 

**Table 6.** Comparison of Neonatal Blood Glucose Levels Between Preterm and Full-Term Neonates (Mann-Whitney U Test) ^a^

Time Interval (h)	Preterm (< 37 wk)	Full-Term (≥ 37 wk)	P-Value
**0 (at birth)**	46.8 ± 12.4	58.2 ± 13.7	0.024 ^b^
**1 **	51.3 ± 14.1	56.9 ± 15.2	0.131
**3 **	60.5 ± 16.7	62.8 ± 15.9	0.384
**6 **	68.1 ± 17.5	70.2 ± 16.8	0.276

^a^ Values are expressed as mean ± SD.

^b^ Significant at P < 0.05, using Mann-Whitney U test.

## 5. Discussion

Maternal diabetes is increasingly prevalent, with a meta‐analysis in Middle Eastern and North African countries reporting a rise in GDM prevalence from 10.6% before 2009 to 14% from 2010 onward (15). The findings underscore the heightened risk of neonatal hypoglycemia in this population, particularly among infants of mothers with poorly controlled diabetes. Aligning with these results, Lorenc and Otto-Buczkowska reported that maternal hyperglycemia significantly influences fetal insulin production, leading to neonatal hypoglycemia through a series of physiological mechanisms (16). Also, Zhang et al. (17) reported a 19.57% hypoglycemia incidence among neonates of mothers with DM.

Our observations regarding the impact of maternal diabetes subtype on neonatal glycemia are broadly consistent with the findings of Kapustin et al. (18). In both studies, infants of mothers with T1DM experienced the greatest dysregulation of blood glucose, reflecting the chronic and often more volatile metabolic milieu that characterizes insulin-dependent pregnancies. Kapustin et al. (18) additionally reported that T1DM and T2DM pregnancies were associated with higher rates of macrosomia and congenital anomalies; however, this cohort revealed that > 60% of mothers met stringent glycemic targets (HbA1C < 6.0% and two-hour postprandial < 120 mg/dL) and had a low incidence of macrosomia and no structural malformations, despite a similarly high prevalence of early neonatal hypoglycemia (≈ 33% of infants of diabetic mothers). This suggests that intensive prenatal glucose control may mitigate risks of excess fetal growth and mal-development but does not eliminate the propensity for hypoglycemia in the immediate postnatal period, especially among infants of T1DM mothers.

Yamamoto et al. (19) found that neonatal hypoglycemia is common in women with T1DM (28%) and T2DM (18%), but less common in women with GDM (5.0%). In contrast, in this study, T1DM shows a strong significant correlation with neonatal blood glucose levels over time, but T2DM and GDM show non-significant correlations. The discrepancy between the results may be attributed to the varying sample sizes used in each study.

Analyzed data demonstrated that poor glycemic control is associated with lower blood glucose levels at birth due to maternal hyperglycemia or impaired insulin secretion during pregnancy. This suggests that early neonatal intervention is crucial in managing hypoglycemia and preventing long-term complications. These findings echo previous research that emphasizes the importance of maintaining tight glycemic control during pregnancy, particularly in mothers with GDM (20). Consistent with our results, Gonzalez-Quintero et al. (21) found that > 1/3 of infants in the poorly controlled group tested positive for at least one factor from a composite variable, including macrosomia, hypoglycemia, jaundice, or stillbirth, compared to 24% in the controlled group. Another study noted that poor maternal glycemic control can lead to neonatal hypoglycemia, as the infant's pancreas is exposed to excess maternal glucose levels, leading to an increase in fetal insulin production and a subsequent risk of low blood sugar at birth. However, the borderline significance observed in this study may indicate the need for larger sample sizes or more nuanced classifications of control to more robustly assess the impact of maternal glycemic management on neonatal outcomes (22).

Moreover, Kujur and Devimeenakshi (20) reported that poor maternal glycemic control, particularly during the third trimester, is strongly associated with neonatal hypoglycemia. Conversely, while maternal glycemic control is critical, Muntean et al. (22) suggest that even with optimal management, infants of diabetic mothers may still face adverse outcomes, indicating that other factors may also play a role in neonatal health. Some studies have also shown that moderate glycemic control, though better than poor control, does not always result in optimal neonatal outcomes, possibly due to residual maternal hyperglycemia or variability in insulin therapy. This suggests that maternal glycemic control may need to be optimized beyond just avoiding poor control to achieve the best neonatal blood glucose outcomes (23).

In a classic study of infants of diabetic mothers, Cioccale et al. (24) observed a significant inverse relationship between birth weight and early postnatal blood glucose levels (r≈ -0.21, P < 0.05). Similarly, a significant negative correlation was demonstrated at birth (0 h: r = -0.225, P = 0.021) and at one hour (r = -0.245, P = 0.012), which weakened by three hours (P = 0.148) and disappeared by six hours (P = 0.691). These parallel results underscore that larger neonates, likely due to higher endogenous insulin, are at greater risk of transient hypoglycemia immediately after delivery. Still, blood glucose tends to stabilize within the first six hours of life (25).

Preterm infants are uniquely predisposed to neonatal hypoglycemia because of limited glycogen and fat stores and immature gluconeogenic pathways, which impair their ability to maintain euglycemia in the immediate postnatal period (26). Adamkin (27) identified prematurity as a primary risk factor for hypoglycemia within the first 24 hours of life. In the current study, gestational age was not significantly correlated with blood glucose at birth, 1, or 3 hours (P > 0.05). Still, it demonstrated a slight positive correlation at six hours (r = 0.213, P = 0.030), indicating that more mature infants tend to achieve higher glucose levels later in the transitional period. This pattern suggests that while prematurity increases early hypoglycemia risk, the maturational advantages of term infants, such as more developed metabolic regulation and better feeding tolerance, become evident several hours after birth, consistent with prior mechanistic insights. Also, most participants of this study received oral medication rather than insulin or combination therapy. Interestingly, Cioccale et al. (24) demonstrated that the type of maternal treatment (diet vs. insulin) did not significantly affect neonatal hypoglycemia incidence (22).

While the study offers valuable insights, several limitations must be acknowledged, such as its observational design that limits its ability to establish causality between maternal and neonatal factors and neonatal hypoglycemia. Also, a small sample size for T1DM (n = 3) limits the generalizability of findings in this subgroup. Larger sample sizes and longer follow-up times would help clarify the trends observed in T2DM and GDM.

### 5.1. Conclusions

This study emphasizes the significant impact of T1DM and maternal glycemic control on neonatal blood glucose levels. The findings highlight that preterm infants are more prone to early hypoglycemia, although glucose levels tend to stabilize within the first few hours of life. Birth weight also showed an inverse relationship with early glucose values, indicating that heavier infants may experience transient hypoglycemia due to higher insulin activity. These results underscore the importance of optimal maternal glycemic management during pregnancy and early postnatal monitoring, particularly among preterm and large-for-gestational-age infants. Future studies with larger samples should further clarify these associations and evaluate preventive interventions to improve neonatal glycemic outcomes.

## Data Availability

The raw data used in this study are available from the corresponding author and can be provided upon request.

## References

[1] Centers for Disease Control and Prevention (2024). Diabetes and Women..

[2] Durnwald C, Nathan D, Werner E, Barss V (2021). Gestational diabetes mellitus: Glycemic control and maternal prognosis..

[3] Salehi MR, Ghaemi M, Masoumi S, Azadnajafabad S, Norooznezhad AH, Vahdani FG (2023). Comparative Analysis of Corticosteroid Therapy in Pregnant Women with COVID-19: Evaluating Glycemic Control and Transient Hyperglycemia.. Fertil, Gynecol Androl..

[4] Anjum K (2018). A study of neonatal outcome in infants born to diabetic mothers at a tertiary care hospital.. Int J Contemporary Pediatrics..

[5] Alemu BT, Olayinka O, Baydoun HA, Hoch M, Akpinar-Elci M (2017). Neonatal hypoglycemia in diabetic mothers: A systematic review.. Curr Pediatric Res..

[6] Shyvi Ravindra S, Abdul Rahman A, R. Aithal R, Bhat S, D A (2025). Comparison of Single Intraoperative Dose of Dexamethasone on Glycemic Profile in Postoperative Diabetic and Non-diabetic Patients.. Anesth Pain Med..

[7] Salih JM (2024). Glycemic Profiles and Hypoglycemia Awareness Among Pregnant Women with Gestational and Pre-existing Diabetes Referred to a Tertiary Center in Sulaimaniyah-Iraq in 2024.. Int J Endocrinol Metab..

[8] Mehryar HR, Mohebbei S (2024). Measuring the Incidence of Periodontal Disease in Patients with Diabetes Hospitalized in Imam Khomeini Educational Center of Urmia.. J Arch Military Med..

[9] Metzger BE, Coustan DR, Trimble ER (2019). Hyperglycemia and Adverse Pregnancy Outcomes.. Clin Chem..

[10] Osman KS, Albakoush AM, Azab AE, Shahpoon SA, Alhadi M, Hadoud KBA (2025). Prevalence of Gestational Diabetes Mellitus among Pregnant Women at Zawia Region.. Prevalence..

[11] Edwards T, Harding JE (2020). Clinical Aspects of Neonatal Hypoglycemia: A Mini Review.. Front Pediatr..

[12] Alsaleem M, Saadeh L, Kamat D (2019). Neonatal Hypoglycemia: A Review.. Clin Pediatr (Phila)..

[13] Theisler C (2023). Diabetes Mellitus Type 2 (DM2)/Adult Onset Diabetes..

[14] Tappis H, Lak R, Alhilfi R, Zangana AH, Wadi F, Hipgrave D (2023). Quality of maternal and newborn health care at private hospitals in Iraq: a cross-sectional study.. BMC Pregnancy Childbirth..

[15] Al-Rifai RH, Abdo NM, Paulo MS, Saha S, Ahmed LA (2021). Prevalence of Gestational Diabetes Mellitus in the Middle East and North Africa, 2000-2019: A Systematic Review, Meta-Analysis, and Meta-Regression.. Front Endocrinol (Lausanne)..

[16] Lorenc A, Otto-Buczkowska E (2018). Maternal Diabetes Mellitus – Risk Factor for Fetus and Infant.. J Endocrinol Diabet..

[17] Zhang X, Rehemutula R, Jin H, Teng Y, Ma J, Mei S (2024). Risk Factors for Hypoglycemia Among Neonates: A Prospective Cohort Study Among Pregnant People With Gestational Diabetes Mellitus.. J Perinat Neonatal Nurs..

[18] Kapustin RV, Kopteeva EV, Alekseenkova EN, Kovalchuk-Kovalevskaya OV, Rybachek AV, Arzhanova ON (2024). Neonatal outcomes in women with diabetes mellitus — analysis of DAPSY data.. J Obstetrics Women's Dis..

[19] Yamamoto JM, Donovan LE, Mohammad K, Wood SL (2020). Severe neonatal hypoglycaemia and intrapartum glycaemic control in pregnancies complicated by type 1, type 2 and gestational diabetes.. Diabet Med..

[20] Kujur AK, Devimeenakshi K (2022). Correlation of glycosylated hemoglobin and macrosomia in infants of diabetic mothers.. Perinatal J..

[21] Gonzalez-Quintero VH, Istwan NB, Rhea DJ, Rodriguez LI, Cotter A, Carter J (2007). The impact of glycemic control on neonatal outcome in singleton pregnancies complicated by gestational diabetes.. Diabetes Care..

[22] Muntean M, Prelipcean I, Racean MA, Cucerea M, Fagarasan A, David CT (2023). Optimally Controlled Diabetes and Its Influence on Neonatal Outcomes at a Level II Center: A Study on Infants Born to Diabetic Mothers.. Medicina (Kaunas)..

[23] Sharma A, Davis A, Shekhawat PS (2017). Hypoglycemia in the preterm neonate: etiopathogenesis, diagnosis, management and long-term outcomes.. Transl Pediatr..

[24] Cioccale A, Brener Dik P, Galletti MF, Mariani G, Lupo E (2022). Hipoglucemia neonatal en hijos de madres con diabetes mellitus gestacional. Comparación de la incidencia según el tratamiento materno.. Arch Argentinos Pediatria..

[25] Abramowski A, Ward R, Hamdan AH (2025). Neonatal Hypoglycemia..

[26] Rozance PJ (2014). Update on neonatal hypoglycemia.. Curr Opin Endocrinol Diabetes Obes..

[27] Adamkin DH (2015). Metabolic screening and postnatal glucose homeostasis in the newborn.. Pediatr Clin North Am..

